# Extracellular Xylanolytic and Pectinolytic Hydrolase Production by *Aspergillus flavus* Isolates Contributes to Crop Invasion

**DOI:** 10.3390/toxins7083257

**Published:** 2015-08-19

**Authors:** Jay E. Mellon

**Affiliations:** Agricultural Research Service, Southern Regional Research Center, U. S. Department of Agriculture, 1100 Robert E. Lee Blvd., New Orleans, LA 70124, USA; E-Mail: jay.mellon@ars.usda.gov; Tel.: +1-504-286-4358; Fax: +1-504-286-4419

**Keywords:** *Aspergillus flavus*, atoxigenic isolate, biocontrol agent, xylanolytic hydrolases, pectinolytic hydrolases

## Abstract

Several atoxigenic *Aspergillus flavus* isolates, including some being used as biocontrol agents, and one toxigenic isolate were surveyed for the ability to produce extracellular xylanolytic and pectinolytic hydrolases. All of the tested isolates displayed good production of endoxylanases when grown on a medium utilizing larch xylan as a sole carbon substrate. Four of the tested isolates produced reasonably high levels of esterase activity, while the atoxigenic biocontrol agent NRRL 21882 isolate esterase level was significantly lower than the others. Atoxigenic *A. flavus* isolates 19, 22, K49, AF36 (the latter two are biocontrol agents) and toxigenic AF13 produced copious levels of pectinolytic activity when grown on a pectin medium. The pectinolytic activity levels of the atoxigenic *A. flavus* 17 and NRRL 21882 isolates were significantly lower than the other tested isolates. In addition, *A. flavus* isolates that displayed high levels of pectinolytic activity in the plate assay produced high levels of endopolygalacturonase (pectinase) P2c, as ascertained by isoelectric focusing electrophoresis. Isolate NRRL 21882 displayed low levels of both pectinase P2c and pectin methyl esterase. *A. flavus* appears capable of producing these hydrolytic enzymes irrespective of aflatoxin production. This ability of atoxigenic isolates to produce xylanolytic and pectinolytic hydrolases mimics that of toxigenic isolates and, therefore, contributes to the ability of atoxigenic isolates to occupy the same niche as *A. flavus* toxigenic isolates.

## 1. Introduction

*Aspergillus flavus* is a ubiquitous saprophytic fungus commonly found in tropical and semitropical climes [1]. This fungus is also capable of opportunistic pathogenesis of oilseed commodities (maize, cotton, peanuts, tree nuts). It has agronomic significance due to the potential fungal contamination of these commodities with the concomitant production of the potent carcinogen aflatoxin B_1_ [2]. Commodity contamination limits are strictly enforced by regulatory agencies (Food & Drug Administration) of the United States, resulting in severe economic losses when the commodities with excessive levels of aflatoxin B_1_ contamination have to be destroyed [3].

One of the major thrusts for control of aflatoxin contamination has evolved from the use of atoxigenic isolates of *A. flavus*. Simply stated, this control strategy employs the introduction of an atoxigenic isolate, which is thought to competitively exclude the proliferation of toxigenic isolates, thereby reducing the levels of aflatoxin contamination. A number of examples of this strategy are actively being pursued in field environments in the United States. The first biocontrol agent that was employed in this manner was *A. flavus* isolate AF36 on cotton [4]. Another *A. flavus* biocontrol agent (NRRL 21882) that is commercially available was isolated from peanut fields [5]. More recently, *A. flavus* K49, isolated from maize, has been registered for aflatoxin biocontrol use [6].

In order for an *A. flavus* atoxigenic isolate to be a good candidate as a biocontrol agent, it presumably should demonstrate infection of susceptible host plants. One measure of fungal infection would be the ability of the fungus to penetrate plant wall tissues. Although plant walls are complex structures, in a simplified model, they are comprised of three general classes of polysaccharide wall components. First are the cellulose microfibrils that function as major rod-like structural components. Cellulose is a polymer consisting of thousands of glucose monomers covalently linked in a linear β-D-1,4 configuration. Each microfibril consists of multiple (10–50) cellulose strands. Next, the cellulose microfibrils are immersed in a matrix of xylan and pectic polysaccharides that are quite complex in their composition. This xylan/pectic glycan matrix helps give orientation and structural stability by cross-linking the cellulose microfibrils. Pectic polysaccharides provide a cohesive element that binds plant cells into a tissue structure (middle lamella between cells), and controls cell wall porosity (Ca cross-linking). Pectins are plant-produced methylated polymers of galacturonic acid. Xylan polysaccharides are complex polymers consisting of multiple xylose residues in a linear β linkage. A number of different substituents may be attached to the xylan backbone, including glucuronic acid, arabinose, galactose, ferulic acid and acetyl residues. Composition variability in these different components provides wall tissues with potential functional diversity.

In order to effectively breach plant wall tissues, *A. flavus* isolates that are crop invasive should be capable of hydrolyzing these major plant wall components. *A. flavus* would appear to have ample genetic capacity to secrete hydrolytic activities targeting cellulose (glucans), xylans and pectins [7]. When *A. flavus* was grown on a medium containing larch xylan as the sole carbon substrate, the fungus secreted a 14-kD endoxylanase as the primary xylanolytic activity [8], in addition to multiple other hydrolases. A specific *A. flavus*-produced endopolygalacturonase (pectinase P2c) has been shown to be highly correlated to fungal virulence against cotton [9]. 

In order to evaluate the ability of *A. flavus* atoxigenic isolates to penetrate plant cell walls as an indication of their effectiveness, a survey was undertaken to determine the production of secreted xylanolytic and pectinolytic activities. The collection of atoxigenic *A. flavus* isolates included AF36, K49, NRRL 21882 and 3 atoxigenic isolates from the laboratory of Dr. K. Damann (17, 19, 22; Louisiana State University), as well as the toxigenic L strain isolate *A. flavus* AF13 for comparison purposes. *A. flavus* L strain isolates produce some sclerotia with diameters greater than 400 μm [10]. This toxigenic isolate was chosen because it is an aggressive toxin producer that has proven to be capable of successful crop invasion, and is metabolically adaptive. Results of this investigation are presented here.

## 2. Results

Biomass production for the tested *A. flavus* isolates fell within the expected range for fungal growth utilizing xylan as a sole carbon substrate. *A. flavus* cultures produced similar biomass levels to those observed in xylan medium when grown on a medium containing an easily accessible saccharide (e.g. sucrose) and do not constitutively secrete xylanases or pectinases under these conditions. There were no significant differences observed for biomass production among the atoxigenic isolates and the toxigenic *A. flavus* isolate (AF13) (Table 1), though the K49 isolate biomass yield appeared to be somewhat low.

All of the tested *A. flavus* isolates produced ample levels of xylanolytic hydrolases, as shown by the xylanase activity data (Table 1). The observed xylanase data revealed no significant differences among the tested fungal isolates. The esterase activity data did reveal some differences (Table 1). Isolates 22, K49, AF36 and AF13 all exhibited high esterase activity, with no significant differences between them. Isolates 17 and 19 trended towards lower esterase levels, and the NRRL 21882 isolate yielded esterase levels significantly lower than the other isolates (Table 1).

**Table 1:** Xylanolytic hydrolases secreted by atoxigenic and toxigenic *A. flavus* isolates.

*A. flavus* Isolate	Dry Weight ^a^	Xylanase Activity ^b^	Esterase Activity ^c^
17	87 ± 21	3.60 ± 0 ^d^	0.0902 ± 0.008
19	110 ± 26	3.38 ± 0.38	0.0801 ± 0.009
22	83 ± 45	3.60 ± 0 ^d^	0.183 ± 0.018
NRRL 21882	90 ± 10	3.60 ± 0 ^d^	0.0636 ± 0.011
K49	60 ± 10	3.15 ± 0.18	0.146 ± 0.047
AF36	90 ± 20	3.27 ± 0.33	0.129 ± 0.041
AF13	83 ± 6	3.26 ± 0 ^d^	0.157 ± 0.01

^a^ Mean dry weights expressed in mg ± standard deviation (*n* = 3). ^b^ Mean xylanase activity expressed in cm^2^ (total area less well area) ± standard deviation (*n* = 3). Details given in text. ^c^ Mean esterase activity expressed as Δ A_400nm/min_ ± standard deviation (*n* = 3). Details given in text. ^d^ All replicates yielded identical values.

Biomass production from the tested *A. flavus* isolates grown on the pectin medium revealed no significant differences (Table 2). However, there were significant differences in pectinolytic activity observed between these isolates. Isolates 19, 22, K49, AF36 and AF13 all demonstrated high pectinolytic activity (Table 2). Activity levels of the 17 and NRRL 21882 isolates were significantly lower than the other tested isolates.

**Table 2:** Pectinolytic activity expressed by atoxigenic and toxigenic *A. flavus* isolates.

*A. flavus* Isolate	Dry Weight ^a^	Pectinolytic Activity ^b^
Control	N.A.	0 ^c^
17	59.3 ± 3.1	1.47 ± 0.81
19	58.0 ± 2.6	5.32 ± 0.24
22	55.0 ± 6.2	5.46 ± 0.24
NRRL 21882	58.7 ± 7.1	2.06 ± 0.39
K49	60.3 ± 1.5	5.32 ± 0.24
AF36	54.7 ± 2.9	5.60 ± 0.43
AF13	57.0 ± 5.3	5.19 ± 0.41

^a^ Mean dry weights expressed in mg ± standard deviation (*n* = 3). ^b^ Mean pectinolytic activity expressed in cm^2^ (total area less well area) ± standard deviation (*n* = 3). Details given in text. ^c^ Well filled with non-inoculated pectin growth medium.

**Figure 1:**
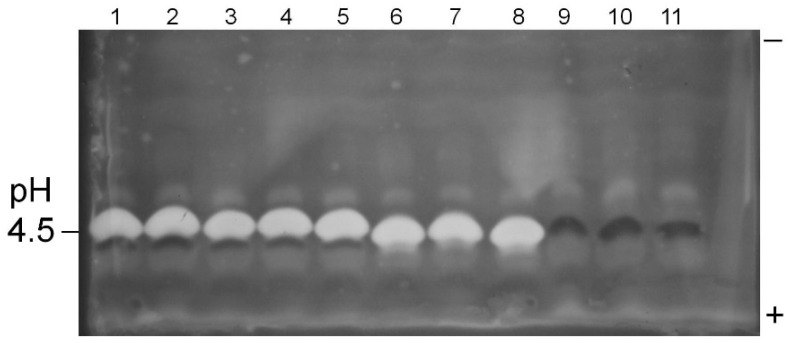
Pectinase P2c/PME profiles of *A. flavus* isolate culture filtrates. Pectinase P2c activity appears as white bands; PME activity appears as dark bands. Samples were prepared as given in text. The polarity orientation of the IEF gel is noted in the right margin; the pH is indicated in the left margin. Lanes 1–2: AF13; lanes 3–5: *A. flavus* 22; lanes 6–8: *A. flavus* 19; lanes 9–11: *A. flavus* 17.

**Figure 2:**
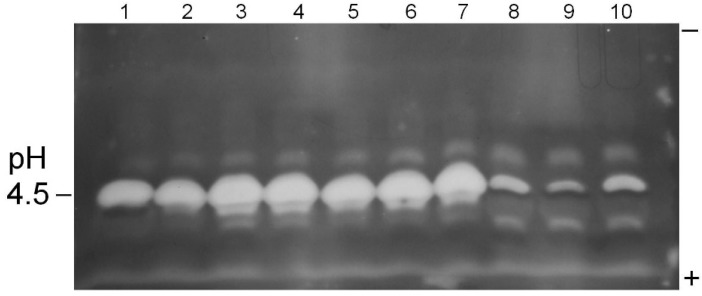
Pectinase P2c/PME profiles of *A. flavus* isolate culture filtrates. Pectinase P2c activity appears as white bands; PME activity appears as dark bands. Samples were prepared as given in text. The polarity orientation of the IEF gel is noted in the right margin; the pH is indicated in the left margin. Lane 1: AF13; lanes 2–4: AF36; lanes 5–7: K49; lanes 8–10: NRRL 21882.

Analysis by isoelectric focusing electrophoresis of the filtrates grown on pectin medium resolved a prominent endopolygalacturonase activity (P2c) from pectin methylesterase (PME) activity. Isolates 19, 22, K49, AF36 and AF13 all expressed high levels of pectinase P2c (Figure 1 and Figure 2; Table 3). The NRRL 21882 isolate secreted significantly lower levels of pectinase P2c, while isolate 17 did not appear to secrete detectable levels of pectinase P2c. The pI value for the observed pectinase P2c bands was 4.45, which is consistent with previous studies [11] and confirmed its identity. Isolates K49 and AF36 also produced a minor pectinase activity band that resolved from P2c (pI slightly more acidic than P2c). In addition, isolate 17 produced significantly higher levels of PME (Table 3; Figure 1) that was not observed in the other tested isolates. 

**Table 3:** Electrophoretic survey of atoxigenic and toxigenic *A. flavus* isolates for pectinase P2c and pectin methylesterase (PME) activities.

*A. flavus* Isolate	P2c ^a^	PME ^a^
17	−	++
19	+++	−
22	+++	+
NRRL 21882	+	+
K49	+++	−
AF36	+++	−
AF13	+++	+

^a^ Abundance of pectinase P2c and PME activities on a scale of – to +++, where – indicates lack of activity and +++ indicates maximal activity.

This “red ring effect” was also noted in the pectinolytic plate assay. Also, all of the tested isolates expressed at least two additional, minor pectinolytic activities (bands) that resolved from the P2c band.

## 3. Discussion

The mode of action of atoxigenic *A. flavus* isolates as biocontrol agents has been investigated to some extent, and a few theories have been put forth. Evidence has been obtained for niche specialization of specific vegetative compatibility groups for *A. flavus* isolates isolated from maize [12]. In addition, the mechanism of *A. flavus*-induced biocontrol appears centered around intraspecific aflatoxin inhibition [13] mediated by a requirement for physical contact (thigmo-regulated) between the toxigenic isolate and atoxigenic isolate [14]. Also, there is evidence for specificity of an atoxigenic strain to effect down-regulation of aflatoxin production in a given toxigenic strain [14].

In this study, it was observed that the atoxigenic *A. flavus* isolates all secreted similar levels of xylanolytic hydrolase activities as the toxigenic isolate when grown on a medium containing larch xylan as the sole carbon substrate (Table 1). *A. flavus* K49 secretes at least two endoxylanases when grown on the same medium [8]. The primary endoxylanase activity is expressed by a 14 kD thermostable protein. A secondary endoxylanase activity is expressed by a 29 kD protein. *A. flavus* AF13, F3W4 and AF36 isolates produce extracellular hydrolase profiles similar to K49 when grown on a xylan medium (data not shown). Since xylanase production is comparable in both atoxigenic and toxigenic strains, this factor probably does not play a critical role in the efficacy of an atoxigenic biocontrol strain. But, this activity may help the atoxigenic biocontrol *A. flavus* strains to compete with toxigenic strains for niche dominance.

Expression of esterase activity differed among isolates in this growth medium. The most probable esterase activity in these preparations is acetylxylan esterase that removes acetyl groups from xylan polysaccharide substrates. This hydrolase activity has previously been identified in similar *A. flavus* medium preparations (unpublished results). In spite of these differences in expressed esterase activities, there were no significant differences observed in biomass production among these isolates grown on the xylan medium. Results support the conclusion that all of these *A. flavus* isolates are capable of digesting plant host hemicellulose wall moieties.

*A. flavus* isolates 19, 22, K49, AF36, and AF13 all exhibited increased levels of pectinolytic activity (Table 2). However, the pectinolytic levels produced by the 17 and NRRL 21882 isolates were significantly lower than those of the other tested isolates. Despite these differences in pectinolytic activity, no differences were observed in biomass production by these same isolates grown on the pectin medium. It is possible that fungal cultures grown for five days may have a sufficient period for pectin digestion for isolates relatively deficient in P2c pectinase production, or pectin lyase activities may be present. These same atoxigenic isolates (17, NRRL 21882) produced low levels of pectinolytic activities that were electrophoretically distinct from P2c. However, pectinase P2c expression levels may correlate to the rate at which a given isolate can macerate host pectin moieties, and thus, its relative ability to penetrate plant cell walls. Previous results have shown that *A. flavus* strains lacking the capacity for P2c production acquire increased ability to cause intercarpellary membrane damage and invade adjacent cotton locules when transformed with the gene for P2c expression [15].

The electrophoretic survey provided additional details of results obtained in the pectinolytic plate assay. Isolates producing a prominent clear zone in the plate assay showed high expression levels of the P2c endopolygalacturonase (Figure 1 and Figure 2; Table 3). Atoxigenic isolate 17, which displayed low activity in the pectinolytic plate assay, did not express detectable levels of pectinase P2c, but did express moderate PME levels. The NRRL 21882 isolate expressed low levels of both pectinase P2c and PME. Previous work has indicated a lack of correlation between PME expression and *A. flavus* isolate virulence against plant hosts. A previous study has shown a lack of correlation between isolate pectinase P2c expression and aflatoxin contamination in cottonseed [16]. Thus, there are probably multiple factors, in addition to P2c production, involved in aflatoxin contamination of oilseed crops.

Results obtained from this study indicate that all of the tested *A. flavus* isolates are fully capable of digesting xylan components of plant cell walls, with more variability in pectin degradation. Such attributes are expected to contribute to the ability of a given *A. flavus* biocontrol strain to effectively compete with toxigenic strains for potential host substrates and thereby occupy the same physical niche. The reduced capacity of *A. flavus* isolates 17 and NRRL 21882 for pectinase P2c production may indicate that these isolates have a reduced ability for plant tissue maceration, and thus, potentially may be less effective as biocontrol agents than the other atoxigenic isolates tested. However, NRRL 21882 (Afla-Guard) is generally regarded as an effective biocontrol agent for controlling aflatoxin contamination of peanuts. From this study, it is evident that *A. flavus* biocontrol efficacy seems to be related to factors other than simply production of P2c pectinase which has been considered the predominant factor thus far.

## 4. Materials and Methods

### 4.1. Biological Materials

*Aspergillus flavus* isolates AF13 and AF36 were isolated from soil (cotton field) and cottonseed, respectively [17]. NRRL 21882 was isolated from peanut [5] and is commercially available as Afla-Guard from Syngenta Crop Protection (Greensboro, NC, USA). *A. flavus* K49 was isolated from maize and was a gift of Dr. H. K. Abbas (USDA, ARS, NBCL). Atoxigenic *A. flavus* isolates 17, 19, and 22 were also isolated from maize and were a gift from the laboratory of Dr. K. Damann. All isolates were maintained on 5/2 agar (5% V-8 vegetable juice, 2% agar, pH 5.2) to ascertain purity before use. Agar plugs were submerged in sterile deionized water to attain conidial suspensions (10^6^ spores/mL) that were used for fungal inoculations. Larch xylan, citrus pectin, Remazol Brilliant Blue R, and *p*-nitrophenyl acetate were obtained from Sigma Chemical Co. (St. Louis, MO, USA).

### 4.2. Fungal Incubations 

A chemically defined growth medium containing insoluble larch xylan (0.25 g per 20 mL medium) as a carbon source and sodium nitrate (3 g/L) as a nitrogen source [8] was used for fungal growth in order to ascertain secreted xylanolytic activities. Culture flasks (50-mL) containing 20 mL of liquid medium were inoculated with 100 μL of conidial suspension and incubated at 30 °C for 5 days in a shaking incubator (150 rpm, dark). Fermentation cultures were processed by qualitative filtration *in vacuo* (Whatman no. 4 filter). Filtrates were centrifuged 10,000× *g* for 30 min, and supernatants were filter sterilized (0.22 μm) before storage at 4 °C. Fungal biomass (mycelial pellets) on filter paper were dried at 60 °C for 24 h before dry weights were recorded. Each treatment was replicated three times. 

A defined medium containing 0.5% pectin (*w/v*) was utilized to ascertain pectinolytic hydrolase production by these fungal isolates [16]. Culture flasks (50-mL) containing 20 mL of liquid medium were inoculated with 100 μL of spore suspension and incubated at 30 °C for 5 days in a shaking incubator (150 rpm, dark). Fungal cultures were subjected to qualitative filtration *in vacuo*, as above. Filtrates were filter sterilized (0.22 μm) before storage at 4 °C. Fungal biomass was determined after drying at 60 °C for 24 h. Each treatment was replicated three times.

### 4.3. Xylanolytic Activity Assay 

Xylanase activity was observed by means of a semi-quantitative radial diffusion assay in a medium containing 0.05% (*w/v*) Remazol Brilliant Blue R (RBB)-xylan, as previously described [8]. Active preparations produce a circular clear zone against a blue background. Activity level is reported as the area of the RBB-xylan digestion zone (total area less well area).

### 4.4. Esterase Activity Assay 

Esterase activity is based on the hydrolysis of *p*-nitrophenylacetate, which releases the chromophore *p*-nitrophenol (A_400nm_). The *p*-nitrophenylacetate substrate was dissolved in dimethylsulfoxide to give a 40 mM stock solution. Sterile xylan culture filtrate was utilized as the enzyme preparation. A typical reaction mixture contained 0.90 mL of 20 mM Tris-HCl, pH 7.8, 0.05 mL *p*-nitrophenylacetate and 0.05 mL of culture filtrate. Final concentration of dimethylsulfoxide was 5% (*v/v*) and was needed for substrate solubility. Final concentration of *p*-nitrophenylacetate was 2 mM. In these assay conditions, a background, non-enzymatic hydrolysis of the substrate occurred. Thus, each reaction mixture was carried out in comparison to a blank (dual beam spectrophotometer) that consisted of 2 mM *p*-nitrophenyl acetate in 20 mM Tris buffer. Reaction mixtures were monitored for A_400nm_ for 10 min; enzyme kinetics were linear during this period. Esterase activity is reported as the increase in A_400nm_ per min.

### 4.5. Pectinolytic Activity Assay 

Pectinolytic activity was determined using a semi-quantitative radial diffusion assay in a medium containing 0.5% pectin (*w/v*), 1.0% agarose (*w/v*), 50 mM potassium acetate (pH 5.2), and 10 mM EDTA (in 9-cm disposable Petri dish). Digestion of the pectin medium produced a circular expanding clear zone (white) against a red background. Wells, 5 mm in diameter, were cut into the gel medium and filled with 40 μL of sample solution. Samples were prepared by concentrating pectin culture filtrates 20-fold in centrifuge concentrators (Centricon, mol. wt. cutoff, 10 kD; Amicon, Beverly, MA, USA). Assay plates were incubated at 37 °C (dark) for 18 h, and then stained with aqueous 0.1% ruthenium red solution (*w/v*; Sigma) for 15 min, followed by destaining in deionized water. The diameter of each digestion zone was measured; activity level is reported as area of pectin digestion zone (total area less well area).

### 4.6. Electrophoresis

Samples for electrophoresis utilized the same concentrated pectin medium filtrates previously described (above). Isoelectric focusing gels (IEF) were performed as previously described [18], except commercial 3.5–9.5 pH gradient gels (GE Healthcare, Piscataway, NJ, USA) were used. Gel regions in contact with the electrolyte strips were removed. The IEF gel was carefully placed onto a bed of medium (2–3 mm thick) of the same composition as the pectinolytic assay plates (above). The gel-medium sandwich was incubated at 37 °C for 45 min, after which the gel was peeled off. The incubated medium bed was stained with aqueous 0.1% ruthenium red solution for 15 min and destained with deionized water. Pectin hydrolase bands appeared as white zones against a red background, while pectin methylesterase (PME) bands stained dark red [16]. 
